# Supplementary material for: The Clinical and Psychopathological Profile of Inpatients with Eating Disorders: Comparing Vomiting, Laxative Abuse, and Combined Purging Behaviors
Source: Healthcare (Basel). 2024 Sep 15;12(18):1858. doi: 10.3390/healthcare12181858 (PMC11430986; doi:10.3390/healthcare12181858)
Supplement: Supplementary file 1 [file healthcare-12-01858-s001.zip › healthcare-3154731-supplementary.pdf]

Table S1. Diagnostic subgroups composition of the clinical sample.

ED inpatients	N	%
AN-R	169	56.0
AN-BP	80	26.5
BN	53	17.5

Legend: ED = eating disorders; N = number of individuals; % = percentage; AN-R = Anorexia Nervosa Restricting subtype; AN-BP = Anorexia Nervosa binge-purging subtype; BN = Bulimia Nervosa.

Table S2. X^2 between Eating disorder diagnosis and purging behaviors.

ED inpatients	AN-R<br>N (%)	AN-BP<br>N (%)	BN<br>N (%)
No purging	162 (94.6)	3 (1.8)	6 (3.6)
Vomiting	1 (1.2)*	53 (64.7)	28 (34.1)
Laxative abuse	6 (30.0)*	5 (25.0)	9 (45.0)
Vomiting + laxative abuse	0 (.0)	19 (65.5)	10 (34.5)

Legend: ED = eating disorders; N = number of individuals; % = percentage; AN-R = Anorexia Nervosa Restricting subtype; AN-BP = Anorexia Nervosa binge-purging subtype; BN = Bulimia Nervosa. *= the laxative use is sporadic and does not reach the diagnostic threshold for AN-BP

Table S3. Gender distribution in the clinical sample.

ED inpatients	N	%
Female	286	94.7
Male	16	5.3
Not provided	1	.0

Legend: ED = eating disorders; N = number of individuals; % = percentage.

Table S4. Ethnic composition of the clinical sample.

ED inpatients	N	%
Caucasic	300	99.3
Other	2	.7

Legend: ED = eating disorders; N = number of individuals; % = percentage.

Table S5. Housing solutions of the persons in the clinical sample.

ED inpatients	N	%
With their parents	240	79.5
Independent	56	18.5
Other	6	2.0

Legend: ED = eating disorders; N = number of individuals; % = percentage.

Table S6. Marital status of the individuals in the clinical sample.

ED inpatients	N	%
Single	234	77.5
Engaged	47	15.6
Married/Cohabitant	16	5.3
Divorced	5	1.6

Legend: ED = eating disorders; N = number of individuals; % = percentage.

Table S7. Type of access to the ED Unit.
*

Origin	N	%
Home	181	59.9
ER	43	14.2
Day Hospital	35	11.6

Transfer from units outside Turin	16	5.4
Transfer from psychiatric unit	15	5.0
Transfer from same hospital internal medicine unit	6	2.0
Transfer from other hospital internal medicine unit	3	1.0
Transfer from intensive care unit	1	.3
Community	1	.3
Nursing home	1	.3

Legend: ED = eating disorders; N = number of individuals; % = percentage; ER = emergency room
